# A Phenotarget Approach for Identifying an Alkaloid Interacting with the Tuberculosis Protein Rv1466

**DOI:** 10.3390/md18030149

**Published:** 2020-03-05

**Authors:** Yan Xie, Yunjiang Feng, Angela Di Capua, Tin Mak, Garry W. Buchko, Peter J. Myler, Miaomiao Liu, Ronald J. Quinn

**Affiliations:** 1 Griffith Institute for Drug Discovery, Griffith University, Brisbane, Queensland 4111, Australia; yan.xie4@griffithuni.edu.au (Y.X.); Y.Feng@griffith.edu.au (Y.F.); a.dicapua@griffith.edu.au (A.D.C.); t.mak@griffith.edu.au (T.M.); miaomiao.liu@griffith.edu.au (M.L.); 2 Guangxi Key Laboratory of Efficacy Study on Chinese Materia Medica, Guangxi University of Chinese Medicine, Nanning, 530200, China; 3 Earth and Biological Sciences Directorate, Pacific Northwest National Laboratory, Richland, WA 99354, USA; garry.buchko@pnnl.gov; 4 School of Molecular Biosciences, Washington State University, Pullman, WA 99164, USA; 5 Center for Global Infectious Disease Research, Seattle Children’s Research Institute, Seattle, WA 98109-5219, USA; 6 Departments of Pediatrics, Global Health, and Biomedical Informatics & Medical Education, University of Washington, Seattle, WA 98195, USA

**Keywords:** PhenoTarget approach, MRMS, protein-ligand complex, polycarpine

## Abstract

In recent years, there has been a revival of interest in phenotypic-based drug discovery (PDD) due to target-based drug discovery (TDD) falling below expectations. Both PDD and TDD have their unique advantages and should be used as complementary methods in drug discovery. The PhenoTarget approach combines the strengths of the PDD and TDD approaches. Phenotypic screening is conducted initially to detect cellular active components and the hits are then screened against a panel of putative targets. This PhenoTarget protocol can be equally applied to pure compound libraries as well as natural product fractions. Here we described the use of the PhenoTarget approach to identify an anti-tuberculosis lead compound. Fractions from *Polycarpa aurata* were identified with activity against *Mycobacterium tuberculosis* H37Rv. Native magnetic resonance mass spectrometry (MRMS) against a panel of 37 proteins from *Mycobacterium* proteomes showed that a fraction from a 95% ethanol re-extraction specifically formed a protein-ligand complex with Rv1466, a putative uncharacterized *Mycobacterium tuberculosis* protein. The natural product responsible was isolated and characterized to be polycarpine. The molecular weight of the ligand bound to Rv1466, 233 Da, was half the molecular weight of polycarpine less one proton, indicating that polycarpine formed a covalent bond with Rv1466.

## 1. Introduction

**Figure 1:**
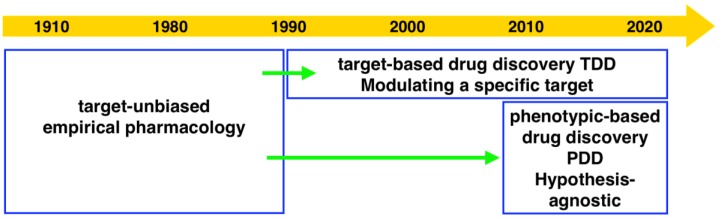
The evolution of technologies and screening strategies in drug discovery from 1910 to 2020. Adapted from [1,2].

Drug discovery research and development has experienced two periods with different centric strategies, namely phenotypic-based drug discovery (PDD) and target-based drug discovery (TDD) (Figure 1). Commonly PDD refers to an approach without prior knowledge of the target. In phenotypic-based screening, compounds that modify a phenotype to generate a positive outcome in cell culture or in a whole organism are identified. TDD examines a specific drug target which is hypothesized to play an important role in disease.

Before the 1980s, the era without recombinant DNA technology, PDD was the primary approach in drug discovery. Most drugs at that time were discovered serendipitously by phenotypic assays in live animals or isolated tissues [3]. Examples are penicillin isolated from a *Penicillium* species in 1928 [2,4] and ivermectin isolated from *Streptomyces avermitilis* in 1975 [5]. From the 1990s, the development of genomics allowed the identification of drug target proteins. The advent of high-throughput screening and combinatorial libraries enabled the screening of target proteins in high throughput. Due to the development of technologies including X-ray crystallography, computational modeling and screening (virtual docking), visualization of the interaction of target protein and compound greatly facilitated the later stage of structure-based development. The development of these technologies appealed to the pharmaceutical industry and academic researchers who then switched to focus on TDD during the last three decades.

In the era that mainly focused on TDD, the total number of new molecular entities (NMEs) and new biologics approved by the Food and Drug Administration was far below expectations [6]. The timeline of cumulative NME approvals from 1950 to 2008 was contributed to by the three most productive companies in the industry and showed almost straight lines, indicating that productivity continued at a constant rate for almost 60 years [1]. The introduction of new molecular biology tools such as recombinant DNA technology, deep sequencing, mining of Expressed Sequence Tagged (cDNA) libraries and the draft human genome did not facilitate drug innovation as expected. This was also indicated in a later NMEs analysis with a timeline spanning over five years to 2013 [7]. More dishearteningly, while the number of NMEs per year has remained relatively constant for the past four decades, the investment in pharmaceutical research and development (R&D) has increased dramatically to over 50 billion USD per year. Today the number of NMEs launched per billion dollars of investment is well below the return for an equivalent billion-dollar investment 50 years ago [1]. The asymmetrical output raises questions about the limitation of the popular target centric strategy of R&D in recent decades.

Consequently, there is a revival of interest in PDD. A significant analysis by Swinney demonstrated that the majority of NMEs approved by the FDA during the 10-year period between 1999 and 2008 were discovered using phenotypic assays, where 28 came from phenotypic screening approaches and 17 came from target-based approaches [8]. It was suggested that the lack of successful NMEs in the post-genomic era was mainly due to the limited use of phenotypic screening. Because an organism is a complex biological system, a simplified single protein assay may not efficiently represent the disease pathogenesis [8] The effects on a single protein may not be translated to meaningful therapeutic efficacy. Conversely, phenotypic screening is an approach for unbiased targets. Phenotypic-based screening is, therefore, being reconsidered for screening compounds due to the realization that results for a single target protein may not fully correlate in the context of a complex biological process [9]. Phenotypic screening also holds the unique promise to uncover new mechanisms of action for currently untreated diseases such as rare diseases and/or neglected diseases [2].

The current phenotypic based approach should not be regarded as a step back to the classical phenotypic screening but as a new discipline [7]. Currently, in vivo and in vitro approaches are involved in phenotypic screening, of which in vitro cell-based phenotypic assays can be easily adapted to a high-throughput format for automated phenotypic analysis. New technologies such as gene expression, genetic modifier screening, resistance mutation and computational inference are increasingly being applied in phenotypic screening. These sophisticated phenotypic screening methodologies enhance the identification of novel compounds as well as their mechanism of action [10].

Target identification is a crucial part of drug discovery. Most large pharmaceutical companies strongly recommend target identification because failing to assign the mechanism of action is frequently regarded as a major risk factor for clinical development and regulatory approval [8]. Even with the most advanced phenotypic screens, in most cases, it is still difficult to determine the mechanism of action. Target identification is the key value of TDD [6]. With the target protein in hand, detailed drug-protein characterization is possible and this provides a better understanding of structure-activity relationships, which is a challenge in phenotypic screening without a known target [11].

Considering the strengths and weaknesses of PDD and TDD, these two approaches should not be treated monolithically but as complementary approaches that can work together to increase the productivity of drug discovery and development. This article describes an approach that combines PDD and TDD: PhenoTarget screening to identify active compounds from natural resources. As a proof of principle, we apply this combination approach to probe fractions of natural product libraries for compounds active against *Mycobacterium tuberculosis* H37Rv, the etiological agent responsible for tuberculosis (TB). The active fraction was then screened against a panel of 37 unique mycobacterial proteins to identify the potential compound and protein responsible for the activity against *M. tuberculosis*.

## 2. Results

**Figure 2:**
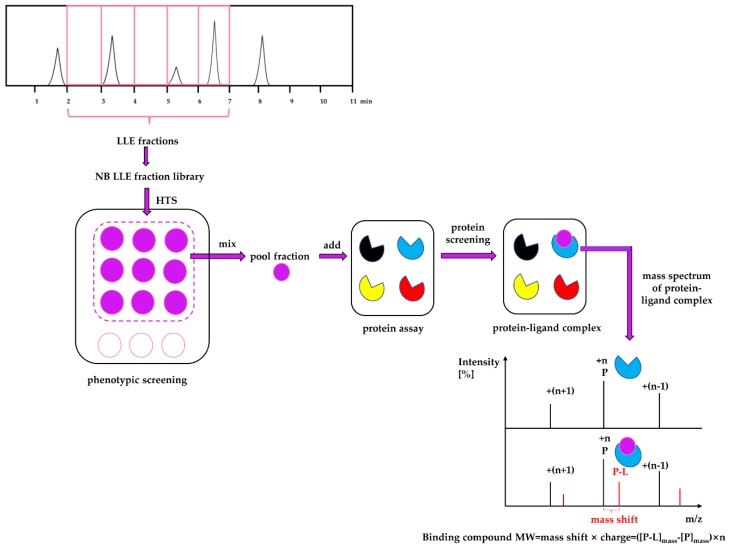
The cascade of PhenoTarget screening for identifying lead compounds and target proteins is shown. The Nature Bank (NB) lead-like enhanced (LLE) fraction library was established following the procedure described by Camp *et al*. [14]. A high throughput phenotypic screening of 202,983 NB LLE fractions against *M. tuberculosis* H37Rv was initially performed. Active fractions with an MIC value of less than 6.1 µge/µL were identified and chosen for protein screening against a panel of 37 putative anti-TB targets from *Mycobacteria* species. To lower sample consumption, especially protein, nine active fractions were pooled (Pool Fractions 1 to 40) and incubated with each of the target proteins. Native mass spectrometry was then used to identify free target protein (P, blue) and protein-ligand (P-L) complexes. The mass shift between the P (black) and the P-L (red) peaks provided the molecular weight of the bound ligand (L, purple) and this facilitated the isolation and identification of the active compound.

A combined approach of phenotypic screening and target screening, which we refer to as a PhenoTarget approach, was used to identify lead compounds against *Mycobacterium tuberculosis* from natural resources. The PhenoTarget approach started from the phenotypic screening of a natural product fraction library to identify fractions that were active in an HTS screen against *M. tuberculosis* H37Rv. Following phenotypic screening, native mass spectrometry was used to simultaneously identify the target protein and the molecular weight of the compound bound to the protein in pooled fractions using a panel of 37 purified mycobacterial proteins. The proteins in the panel were selected because they were all essential enzymes or virulence factors, their three-dimensional structures had been solved by the Seattle Structural Genomics Center for Infectious Diseases (SSGCID); [12,13] and SSGCID was able to supply purified proteins for the PhenoTarget screens. Screening of the pooled fractions against this protein panel of 37 putative anti-TB targets was conducted by a magnetic resonance mass spectrometry (MRMS) system equipped with an automated chip-based Nanomate system. A summary of the PhenoTarget approach is described in Figure 2.

### 2.1. Native Mass Spectrometry

**Figure 3:**
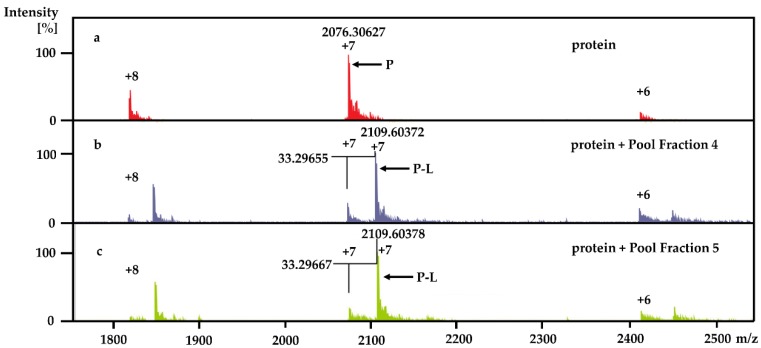
Overlay of the native magnetic resonance mass spectrometry (MRMS) spectra for free Rv1466 (a) and Rv1466 incubated with Pool Fraction 4 (b) and 5 (c). In the spectrum of free Rv1466 (P), clusters of ions corresponding to three different charged states for Rv1466 were observed. The same cluster of ions was observed in the spectra with Pool Fraction 4 and 5 but at lower intensities. Accompanying the ions for free Rv1466 in the spectra with Pool Fractions are clusters of larger intensity ions shifted to high m/z values that correspond to Rv1466-ligand (P-L) complexes. The mass shift for the differently charged cluster pairs (P and P-L) was identical in both Pool Fractions, identifying the molecular weight of the bound ligand: Pool Fraction 4: MW = (2109.60372 – 2076.30627) × 7 = 233 Da; Pool fraction 5: MW = (2109.60378 – 2076.30627) × 7 = 233 Da.

Figure 3 compares the native MRMS spectra for free Rv1466, a protein associated with [Fe–S] complex assembly and repair (a) and NB LLE Pool Fraction 4 (b) and 5 (c). All three spectra contain clusters of ions with three different charged states (+8, +7, and +6). However, the spectra with Pool Fractions 4 and 5 both contain a cluster of ions shifted to high m/z values by identical amounts relative to the free Rv1466 ions. These new ions are also of higher intensity than the free Rv1466 ions and correspond to Rv1466-ligand (P–L) complexes. From the charge and m/z differences between the cluster of P and P–L ions it is possible to obtain the molecular weight of the ligand-bound to Rv1466, 233 Da. Because the ligand that was bound to Rv1466 from Pool Fraction 4 and 5 had the same molecular weight, and likely the same compound, and appeared to have a high affinity for Rv1466 as deduced by the P:P-L intensity ratio, we chose to pursue the identity of this compound.

### 2.2. Target Fraction Confirmation

**Figure 4:**
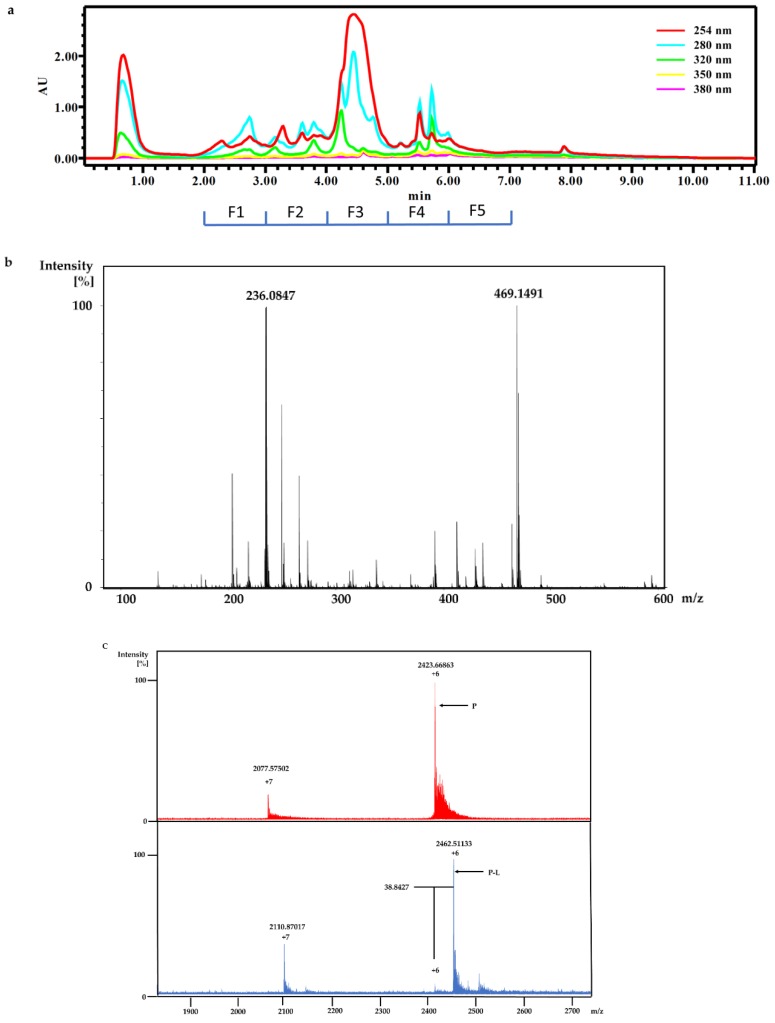
(**a**) Five fractions (Fractions A, B, C, D and E) were collected from a fresh 95% ethanol extraction of *P. aurata*; (**b**) the HRMS analysis of Fraction C identified ions at m/z 236 and 469; (**c**) comparison of the native MRMS spectrum for free Rv1466 (top, red) with the incubation of Rv1466 with Fraction C (bottom, blue). A similar ionization pattern was observed as described in Figure 3 with Pool Fractions 4 and 5. Ions corresponding to free Rv1466 (P) and the Rv1466-ligand complex (P-L) are labeled. Molecular weight of ligand in Fraction C: MW = (2462.51133 – 2423.66863) × 6 = 233 Da.

A feature common to Pool Fractions 4 and 5 was that they contained LLE fractions (LLE-2 and LLE-3, respectively) from the same marine biota, *Polycarpa aurata*, suggesting that a compound from *P. aurata* had interacted with Rv1466. A detailed high-resolution mass spectrometry (HRMS) investigation of all nine fractions from Pool Fraction 5 indicated that no fractions showed an ion at 233 m/z. In this case, MS investigation of the fractions did not confirm the presence of a ligand, so native MS was used to identify the correct fraction for isolation. Thus, five fractions were generated from a re-extraction of *P. aurata* by 95% ethanol and named Fractions A, B, C, D and E (Figure 4a). HRMS analysis of the constituents of Fraction C by a quadrupole-time-of-flight (Q-TOF) mass spectrometer identified an ion at m/z 236 as well as a higher mass ion at m/z 469 corresponding to a 468 + H^+^ ion (Figure 4b). Another round of native MRMS screening confirmed that a compound in Fraction C interacted with Rv1466 and the molecular weight of the bound species was 233 Da (Figure 4c). Because the molecular weight of the ligand bound to Rv1466 was approximately half the molecular weight of the major species in Fraction C, our attention was drawn towards a potentially symmetrical parent compound with a molecular weight of 468 Da as the agent reacting with Rv1466.

### 2.3. Binding Compound Isolation and Structure Elucidation 

**Figure 5:**
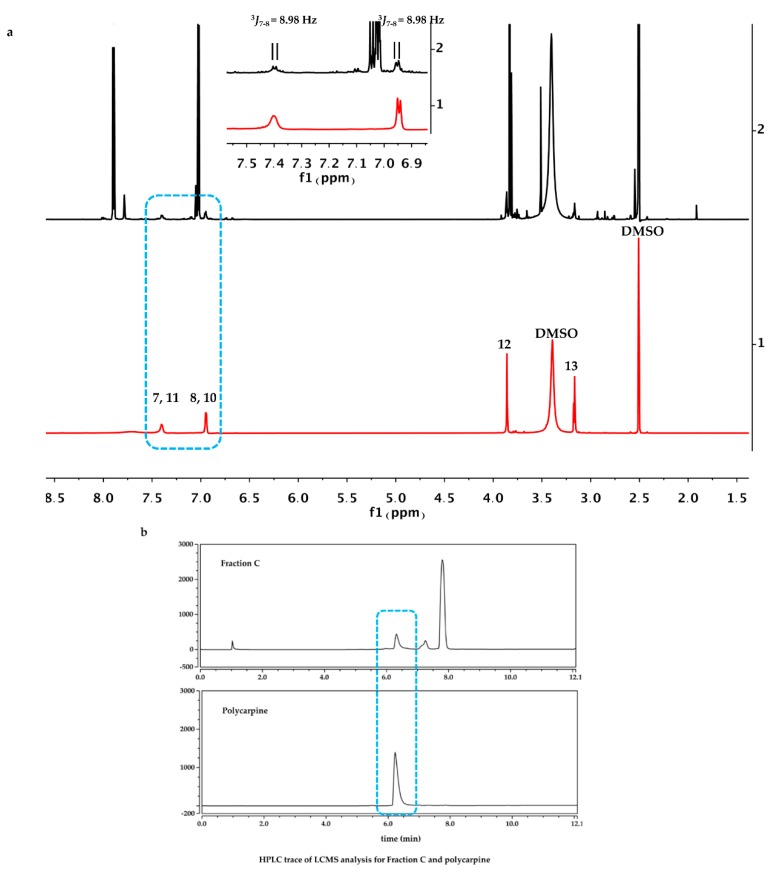

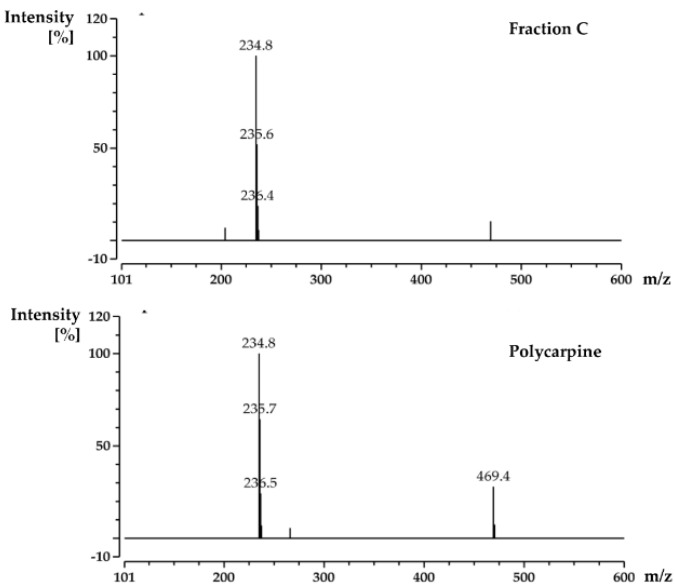
(**a**) ^1^H NMR (recorded in DMSO-*d*_6_) of Fraction C (black, top) and polycarpine (red, bottom). The inset, an expansion of the downfield regions of the spectra circled in blue. (**b**) HPLC analysis of Fraction C (top) and polycarpine (bottom), (**c**) Mass spectral analysis of both HPLC bands circled in a blue rectangle from Fraction C and polycarpine showed an identical ion at 234 Da.

**Figure 6:**
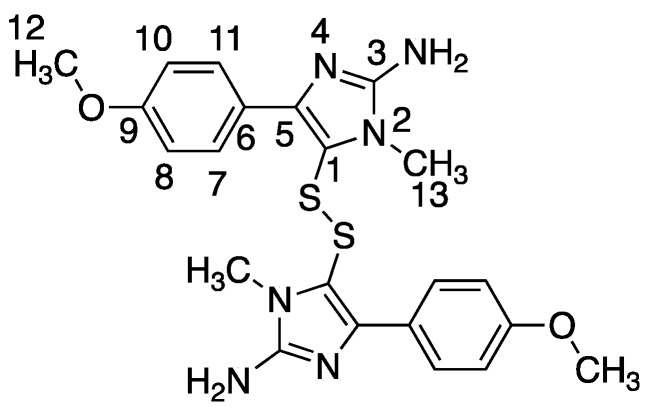
The structure of polycarpine.

Separation of *P. aurata* extracts by reverse phase semi-preparative HPLC (Figure 5) gave a compound identified as polycarpine, C_22_H_24_N_6_O_2_S_2_, a dimeric disulfide alkaloid previously identified from *P. autara* [15] and in a related species, *Polycarpa clavata* [16] (Figure 6). As illustrated in Figure 5a, the resonances observed in the one-dimensional ^1^H spectrum of pure polycarpine (bottom, red) are present in the one-dimensional ^1^H NMR spectrum for Fraction C (top, black). Moreover, pure polycarpine elutes with a retention time identical to a band in the LC profile for Fraction C (Figure 5b). The mass spectra of both bands are identical (Figure 5c) and consistent with a species with a molecular weight of 468 Da.

### 2.4. Pseudo-K_D_ Value Determination of Polycarpine with Rv1466

**Figure 7:**
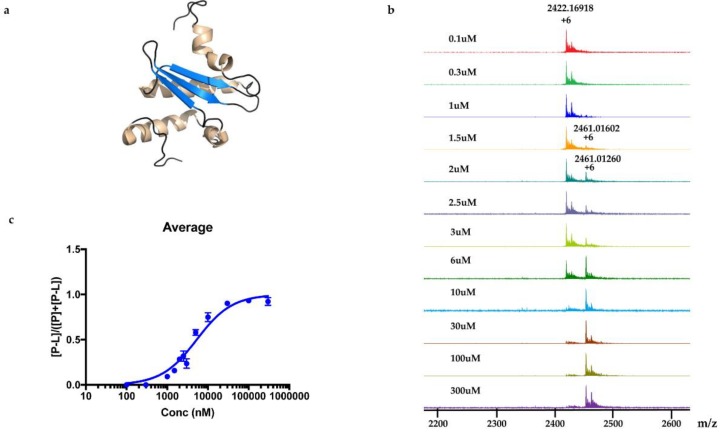
Direct determination of pseudo-*K*_D_ for polycarpine using a dose-response curve. (**a**) Cartoon representation of the Rv1466 structure (5IRD) closest to the average structure in the calculated ensemble. The α-helices and β-strands are colored gold and blue, respectively; (**b**) Overlay of twelve mass spectra of samples containing Rv1466 (9 μM) incubated with varying concentrations of polycarpine (0.1–300 μM); (**c**) The relative mass responses of protein-ligand complex with protein, [P-L]/([P-L]+[P]), plotted against the concentration of polycarpine. The pseudo-*K*_D_ was determined to be 5.29 ± 0.39 μM.

An advantage of native mass spectrometry is that in addition to the qualitative identification of protein-ligand formation, the technique also provides quantitative information on the strength of the interaction [17,18]. This is because it is possible to estimate the dissociation constant, K_D_, from the ratio of free and bound protein observed in the MS chromatograms. It is accomplished by collecting MRMS data at a fixed protein concentration with increasing concentrations of the ligand (Figure 7a). Figure 7b displays twelve mass spectra of samples containing 9 μM Rv1466 and increasing amounts of polycarpine (0.1–300 μM). A ligand concentration was reached where the intensity of the protein-ligand complex reached a plateau. The ratios of the intensity of protein-ligand peak and sum of protein peak and protein-ligand peak were plotted against the concentration of polycarpine (Figure 7c). Using these ratios and Equations 1 and 2, a pseudo-K_D_ of 5.3 ± 0.4 μΜ was calculated for polycarpine binding to Rv1466. A real K_D_ cannot be calculated because after binding a covalent bond forms between the ligand and Rv1466 in a time-dependent manner, pushing the equilibrium to the product.

## 3. Discussion

Using the PhenoTarget approach, a ligand from a natural product fraction library was identified that bound to the *M. tuberculosis* protein Rv1466. The ligand molecular weight from the native MRMS spectrum was 233 Da. The compound was shown to be polycarpine with a molecular mass of 468 Da. The ligand MW of 233 Da was half the molecular weight of polycarpine less one proton, indicating that polycarpine formed a covalent bond with Rv1466. Under mild silica chromatography, the polycarpine disulfide bond has been shown to be cleaved [15,16]. Rv1466 has a single cysteine and the structure of Rv1466 (5IRD) indicates this residue is exposed on the surface of the protein so that it could attack the disulfide bond of polycarpine to form a covalent disulfide linked complex. The polycarpine-Rv1466 pseudo-*K*_D_ was determined to be 5.3 ± 0.4 μM. *P. aurata*, a species of tunicate in the family Styelidae, is rich in alkaloids containing sulfur such as polycarpine, polycarpaurine A, polycarpaurine B and polycarpaurine C [15,16,19,20].

In the PhenoTarget approach, phenotypic screening is conducted initially to detect cellular active components. In our case a natural product fraction library was used, however, this could be equally applied to pure compound libraries. Mass spectrometry using a protein panel of putative targets provided sufficient throughput to analyze the phenotypic hits. Native mass spectrometry offers the further advantage that it can be used for cloned, expressed and purified proteins that lack structural information. This opens the option to explore the proteome of many species, in our case, the *Mycobacterium* proteome. As well as its high sensitivity, high-throughput capability and in time observation of protein-ligand interaction, it can also provide exact mass information of the binding compound [21]. The mass information is useful for isolating the compound from the fraction mixture using mass-guided isolation. In addition, mass spectrometry has been reported as an alternative technique for the quantitative characterization of protein–ligand interactions in solution, that is, the determination of equilibrium constants for protein–ligand association (Ka) or dissociation (Kd = 1/Ka) [22,23]. Among all the mass spectrometers, MRMS provides the highest accuracy and resolution. Nanoelectrospray was used for sample delivery. Compared to conventional electrospray, its benefits include an increased sensitivity signal, lower consumption of ligand and protein, and no sample-to-sample cross-contamination, a faster speed and a higher degree of nozzle-to-nozzle reproducibility [24]. A high degree of nozzle-to-nozzle reproducibility is important for quantitative studies such as *K*_D_ determination. It has been proven that *K*_D_ results from chip-based electrospray are consistent with conventional techniques [17].

While there are advantages in the phenotypic drug discovery approach it should be noted that there are also some disadvantages. Foremost is the procurement of pure protein panels for target identification. For example, the genome of *M. tuberculosis* contains over 4000 genes [25] and our exploratory panel contained only 37 different proteins. This bottleneck can be partly circumvented by using orthologous proteins from related species [26]. Indeed, in addition to *M. tuberculosis* (12 proteins), our mycobacterial panel contained proteins from *Mycobacterium smegmatis* (5), *Mycobacterium abscessus* (4), *Mycobacterium laprae* (3), *Mycobacterium marinum* (3), *Mycobacterium ulcerans* (3), *Mycobacterium fotuitum* (3), *Mycobacterium avium* (2), and *Mycobacterium paratuberculosis* (2). Advantages, on the other hand, lie in the sensitivity and specificity of MRMS for ligand identification.

In summary, while the PhenoTarget approach has some limitations, this article is an example of the powerful potential of the PhenoTarget approach in identifying new lead compounds, from a natural product fraction library, as well as the target protein of the lead compound. In the PhenoTarget approach, phenotypic screening is conducted initially to detect cellular active components. The hits are then screened against a panel of putative targets. This PhenoTarget protocol can be equally applied to pure compound libraries as well as natural product fractions and may prove to be a valuable alternative strategy for developing new intervention therapies.

## 4. Materials and Methods

### 4.1. General Experimental Procedures

NMR spectra were recorded in DMSO-*d*_6_ (δ_H_ 2.50 and δ_C_ 39.5), CD_3_OD (δ_H_ 3.31, 4.78) at 25 °C on the Bruker AVANCE III HDX 800 MHz NMR spectrometer (F$\ddot{a}$llanden, Z$\ddot{u}$rich, Switzerland) equipped with a triple resonance cryoprobe. The low-resolution LC-MS was acquired using Ultimate 3000 RS UHPLC coupled to a Thermo Fisher MSQ Plus single quadruple ESI mass spectrometer (Waltham, MA, USA) with a Thermo Accucore C_18_ column (2.6 μm, 2.1 × 150 mm). High-resolution mass spectra (HRESIMS) were recorded on a Bruker maXis II ETD ESI- qTOF (Bremen, Germany). The HPLC system for LLE fractions for phenotypic screening included a Waters 600 pump (Milford, MA, USA) fitted with a 996-photodiode array detector and Gilson FC204 fraction collector (Middleton, WI, USA). The HPLC system for Fractions A~E from re-extraction by ethanol was the Thermo Ultimate 3000 system (Waltham, MA, USA). Semi-preparative HPLC was also programmed on Thermo Ultimate 3000 with a PDA detector. A Phenomenex C_18_ Monolithic column (5 μm, 4.6 × 100 mm) was used for analytical HPLC; a Thermo Electron Betasil C_18_ column (5 μm, 21.2 × 150 mm) was used for semi-preparative HPLC.

The fraction library (202,983 fractions) was from Nature Bank (Griffith Institute for Drug Discovery, Brisbane, Australia). All the solvents used for extraction, chromatography and MS were Lab-Scan HPLC grade, and the H_2_O was Millipore Milli-Q PF filtered.

Ammonium acetate was purchased from. Sigma-Aldrich (St Louis, O.M., USA). Rv1466 and the other 36 unique proteins in the target panel were supplied by the Seattle Structural Genomics Center for Infectious Diseases (SSGCID, Seattle, WA, USA). These were “well behaved” proteins whose structures have been solved by SSGCID and are publicly available in the PDB. All proteins contained a non-natural, N-terminal tag for purification (MAHHHHHHMGTLEAQTQGPGS-). For protein buffer exchange, Nalgene NAP-5 size G25, from GE Healthcare (Parramatta, NSW, Australia) was used (purchased through Thermo Fisher Scientific Australia Pty Ltd., Scoresby, VIC, Australia). The screening of protein-ligand complexes was conducted using Bruker Daltonics SolariX 12 T Magnetic Resonance Mass Spectrometry (Bruker Daltonics Inc., Billerica, MA, USA) equipped with automatic Nanoelectrospray system (TriVersa NanoMate, Advion Biosciences, Ithaca, NY, USA).

### 4.2. Lead-Like Enhanced Fraction Library

The lead-like enhanced fraction library was prepared as previously described [14].

### 4.3. Phenotypic Screening-Activity against Replicating M. tuberculosis H37Rv

Growth inhibition of *M. tuberculosis* was monitored using a 1 μL of fraction (250 μge/μL) dispensed into each well of a 384-well plate. To this, 40 µL of *M. tuberculosis* ATCC 27294 H37Rv (3–5 × 10^5^/mL in Middlebrook 7H9 broth with 0.05% Tween 80, 10 *v*/*v* ADC and Casamino acids) was added with a Multidrop dispenser. The plates were then incubated at 37 °C for 7 days. A 10 μL solution of Resazurin (20 mg/100 mL diluted 1:1 with 10% Tween 80) was added and incubated further for an additional 24 h at 37 °C for color development. Absorbance was monitored at two wavelengths (575 and 610 nm) using Spectramax and the ratios were determined to calculate the % inhibition. Growth controls in the absence of compound as well as media controls served as inhibition ~0% and −100% respectively.

Single point screen: samples (1 μL) which gave ≥80% inhibition were considered positive. MIC: the least concentration which gave ≥80% inhibition was considered as MIC (start conc. = 1 μL of fraction (250 μge/μL).

### 4.4. Re-extraction and Fractionation

The dry powder of *P. aurata* (1.8 g) was extracted with 95% ethanol (3 × 150 mL) to afford a crude extract (1.48 g). A portion of the crude extract (40 mg) was dissolved *in DMSO-d_6_* (600 μL). Twice injection (2 × 100 μL) was performed for each sample. HPLC separations were performed on a Phenomenex C_18_ Monolithic HPLC column (4.6 × 100 mm) using conditions that consisted of a linear gradient (curve 6) from 90% H_2_O (0.1% TFA)/10% MeOH (0.1% TFA) to 50% H_2_O (0.1% TFA)/50% MeOH (0.1% TFA) in 3 min at a flow rate of 4 mL/min; A convex gradient (curve 6) to 50% H_2_O (0.1% TFA)/50% MeOH (0.1% TFA) at a flow rate of 3 mL/min in 0.01 min, then a linear gradient (curve 5) to 100% MeOH (0.1% TFA) in 3.50 min; A held at 100% MeOH (0.1% TFA) (curve 6) for 1.50 min at a flow rate of 3 mL/min, then held at 100% MeOH (0.1% TFA) (curve 6) with a flow rate increasing to 4 mL/min in 0.01 min; A held at 100% MeOH (0.1% TFA) (curve 6) at a flow rate of 4 mL/min for a further 1 min; A linear gradient (curve 6) back to 90% H_2_O (0.1% TFA)/10% MeOH (0.1% TFA) in 1 min at a flow rate of 4 mL/min, then held at 90% H_2_O (0.1% TFA)/10% MeOH (0.1% TFA) (curve 6) for 2 min at a flow rate of 4 mL/min. The total run time for each injection was 11 min, and 5 fractions were collected between 2.0 and 7.0 min, i.e., Fraction A (time = 2.01-3.00 min), Fraction B (time = 3.01-4.00 min), Fraction C (time = 4.01-5.00 min), Fraction D (time = 5.01-6.00 min) and Fraction E (time = 6.01-7.00 min). Each fraction was dissolved in 200 μL of DMSO-*d*_6_ and was run for ^1^H NMR fingerprint in a 3 mm NMR tube on a Bruker AVANCE III HDX 800 MHz NMR instrument.

### 4.5. Automatic Chip-Based MRMS High Throughtput Screening

Proteins were buffer-exchanged into a suitable volatile buffer (ammonium acetate) under near physiological conditions using size exclusion chromatography prior to MRMS analysis. Depending on the protein, the buffer and its concentration were chosen to obtain the highest sensitivity in the mass spectrometer. Final concentration was 10 μM protein in 10 mM ammonium acetate after buffer change.

All screening samples were prepared fresh on the day for analyzing data to avoid the precipitation or decomposition in the sample.

Each 9 LLE fractions (9 × 1 μL) were combined as a Pool Fraction. Every Pool Fraction was dried, re-suspended in 9 μL MeOH. For the screening of Pool Fractions, 1 μL MeOH solution of pool fraction was mixed with 9 μL of 10 µM protein and then incubated for 1 h at room temperature before being analyzed by MRMS.

For screening the incubation of Fraction C of *P. aurata* with Rv1466-Putative uncharacterized protein, Fraction C was dried and re-suspended in 400 μL MeOH. An aliquot solution (1 μL) was added to protein (9 μL at 10 µM) for 15 min at room temperature and then analyzed by MRMS.

For the screening of pure compound-polycarpine, the stock solution of polycarpine, 3000 μM dissolved in DMSO-*d*_6_, was further diluted to additional varied concentrations between 1–1000 μM for dose-response measurement. Polycarpine solution in DMSO-*d*_6_ was dried and then dissolved in MeOH. A 40 μL sample of these polycarpine solutions was lyophilized and resuspended in 40 μL of MeOH. An aliquot (1 μL) of varied concentration of polycarpine in MeOH was incubated with 9μL of protein (10 µM) for 15 min at room temperature and then analyzed by MRMS.

Experiments were performed on a Bruker SolariX XR 12T MRMS (Bruker Daltonics Inc., Billerica, MA) equipped with an external automated chip-based nanoelectrospray. The ESI mass spectra were recorded in positive mode with a mass range from 294 to 10,000 m/z. Each spectrum was composed of 2 M data points. All the aspects of instrument parameter and data acquisition were controlled by Solarix control software under Windows operating system. Instrument parameters were tuned as follows for Pool Fraction screening: sample flow rate 120 µL/min, nebulizer gas (N_2_) pressure 3, end plate offset voltage 0 V, capillary voltage 1000 V, drying gas (N_2_) flow rate 1.5 L/min, drying gas temperature 100 °C, capillary exit voltage 220 V, skimmer 1 voltage 60 V, collision voltage −5, time of flight 2.1 s, scan 8 and accumulation time 0.5 s. For the screening of Rv1466-Putative uncharacterized protein with Fraction C and polycarpine, six parameters change including skimmer 1 voltage (30 V), collision voltage (−10 V), time of flight (1.8 s), scan (32) and accumulation time (2 s).

All sample solutions were injected by fully automated chip-based nanoelectrospray, which was mounted to the mass spectrometer. A Triversa NanoMate incorporating ESI chip technology (Advion Biosciences, Ithaca, NY) was used as an automatic chip system. The automated chip system consists of a 384-well plate, a rack of 96 disposable, conductive pipette tips, and the chip, which was positioned a few millimeters from the sampling cone. The system was programmed by ChipSoft software (Version 8.3.1, Advion BioSciences), which sequentially picks up a new pipette tip, aspirates 3 μL of sample from the 384-well plate followed by 1.5 µL of air and then delivers to the inlet side of ESI chip. Nanoelectrospray was carried out by applying a 1.60 kV spray voltage and a 1.0 psi gas pressure to the sample in the pipette tip. Sample solutions for screening were transferred into the 384-well plate. For every sample, a fresh tip and nozzle were used, thus preventing cross-contamination of samples. Following sample infusion and MS analysis, the pipet tip was disposed.

When the protein-ligand complex was found, the molecular weight of the binding ligand was estimated from the spectrum using the following equation: MW ligand = Δ m/z × z.

### 4.6. Isolation

Crude ethanolic extract of *P. aurata* (200 mg) was dissolved in MeOH. HPLC separation was conducted on a Thermo Electron Betasil C_18_ column (5 μm, 21.2 × 150 mm) at a flow rate of 9 mL/min: using gradient solvent system (10–100% MeOH in 50 min, 100% MeOH from 50–60min) and 60 fractions were collected by minutes. Fraction 33 and 34 were further purified on the same column using gradient solvent from 30–80% MeOH to afford polycarpine.

Polycarpine was obtained as yellow powder. HRMS (positive mode): m/z 469 [M + H] ^+^ (calculated for C_22_H_25_N_6_O_2_S_2_, 469.1475). ^1^H NMR (CD_3_OD, 800MHz) δ 7.49 (2H, d, *J* = 8.98Hz, H-7/11), 7.03 (2H, d, *J* = 8.98Hz, H-8/10), 3.91 (3H, s, 12-CH_3_), 3.25 (3H, s, 13-CH_3_). Carbon chemical shift was confirmed from 2D NMR, δ 109.8 (C-1), 148.1 (C-3), 137.6 (C-5), 117.6 (C-6), 127.7 (C-7/11), 113.7 (C-8/10), 161.4 (C-9), 54.4 (C-12) and 27.8 (C-13).

### 4.7. Dose-Response Data Analysis

The relative abundances of protein-ligand complex to total protein in the mass spectra correlated to the relative equilibrium concentrations of ligand to total protein in solution. The pseudo *K*_D_ of polycarpine with Rv1466 was determined using the following equations [18]:

(1) $$\frac{\Sigma \;I{\left(P-L\right)}^{n+}/n}{\Sigma \;I\;{\left(P\right)}^{n+}/n\;+\Sigma \;I\;{\left(P-L\right)}^{n+}/n}=\frac{\left[P-L\right]}{{\left[P\right]}_{t}}$$

(2) $$\frac{\Sigma \;I{\left(P-L\right)}^{n+}/n}{\Sigma \;I\;{\left(P\right)}^{n+}/n\;+\Sigma \;I\;{\left(P-L\right)}^{n+}/n}=\frac{{\left[P\right]}_{t}+{\left[L\right]}_{t}\;+K_{D}-\sqrt{\left({\left[P\right]}_{t}+{\left[L\right]}_{t}\;+K_{D}){}^{2}-4{\left[P\right]}_{t}{\left[L\right]}_{t}\right)}}{2{\left[P\right]}_{t}}$$

Experimental relative ratios of protein-ligand complex and total protein ion abundances were plotted against the total concentration of ligand. The pseudo *K*_D_ could be obtained as a parameter of a nonlinear least-squares curve fitting.
